# Phenotypic research on senile osteoporosis caused by SIRT6 deficiency

**DOI:** 10.1038/ijos.2015.57

**Published:** 2016-04-29

**Authors:** De-Mao Zhang, Di-Xin Cui, Ruo-Shi Xu, Ya-Chuan Zhou, Li-Wei Zheng, Peng Liu, Xue-Dong Zhou

**Affiliations:** 1 State Key Laboratory of Oral Diseases, West China Hospital of Stomatology, Sichuan University, Chengdu, China

**Keywords:** ageing, osteoclastogenesis, osteogenesis, osteoporosis

## Abstract

Osteoporosis is a serious public bone metabolic disease. However, the mechanisms underlying bone loss combined with ageing, which is known as senile osteoporosis, remains unknown. Here we show the detailed phenotype of this disease caused by SIRT6 knock out (KO) in mice. To the best of our knowledge, this is the first study to reveal that SIRT6 is expressed in both bone marrow stroma cells and bone-related cells in both mouse and human models, which suggests that SIRT6 is an important regulator in bone metabolism. SIRT6-KO mice exhibit a significant decrease in body weight and remarkable dwarfism. The skeleton of the SIRT6-KO mouse is deficient in cartilage and mineralized bone tissue. Moreover, the osteocalcin concentration in blood is lower, which suggests that bone mass is markedly lost. Besides, the tartrate-resistant acid phosphatase 5b (TRAP5b) concentration is much higher, which suggests that bone resorption is overactive. Both trabecular and cortical bones exhibit severe osteopenia, and the bone mineral density is decreased. Moreover, double-labelling analysis shows that bone formation is much slower. To determine whether SIRT6 directly regulates bone metabolism, we cultured primary bone marrow stromal cells for osteogenesis and osteoclastogenesis separately to avoid indirect interference *in vivo* responses such as inflammation. Taken together, these results show that SIRT6 can directly regulate osteoblast proliferation and differentiation, resulting in attenuation in mineralization. Furthermore, SIRT6 can directly regulate osteoclast differentiation and results in a higher number of small osteoclasts, which may be related to overactive bone resorption.

## Introduction

Osteoporosis is a serious public disease, particularly in China, as China's society is increasingly ageing.^1^ Globally, osteoporosis is the most common bone metabolic disease of the elderly.^2^ There are two subtypes of osteoporosis: type I is called postmenopausal osteoporosis or oestrogen-deficient osteoporosis, and often occurs after female menopause due to a decline in oestrogen levels. Type II is senile osteoporosis (SOP), which usually occurs in females at 65 years of age or in males at 70 years of age. Although great progress has been made in understanding the mechanism underlying oestrogen-deficient osteoporosis within the past several years, it remains unknown how bone loss is caused by ageing, which may be correlated to SIRT6-based mechanisms according to previous research.^3^ Age-related bone loss is associated with ageing in both genders, and there is a greater decrease in bone formation by osteoblasts and a significant increase in bone resorption by osteoclasts,^4^ which consequently results in osteoporosis.

Recent studies have used functional genetic approaches and gene knock out (KO) technology as a new method to study the development of many biological problems in the process of ageing. It is well known that the family of deacetylase sirtuin (SIRT1-7) is involved in ageing and age-related diseases, which provides a new approach to the study of biological diseases, including bone loss in ageing.^3^ It has been reported that the SIRT family is involved in a broad range of biological processes such as DNA repair, neurological protection, and body metabolism. The most recent study has shown that SIRT6, an nicotinamide adenine dinucleotide (NAD)-dependent deacetylase, has a critical role in anti-ageing.^5, 6^ SIRT6 functions as a deacetylase and nucleotidyltransferase, which removes chemicals from acetyl groups from a specific site of the histone, thereby regulating specific gene expression. As reported, SIRT6-deficient mice exhibit a short lifespan and multiple organ ageing, including reduced blood glucose and insulin-like growth factor (IGF) concentrations, increased DNA instability, and bone loss at an early period.^5^ As a histone-H3 lysine deacetylase, SIRT6 can regulate the stability of telomere chromatin and the genetic expression of the nuclear factor-kappa B signalling pathway, which affects lifespan.^7^ SIRT6 is also involved in various biological processes such as inflammation, metabolic balance, and even cancer metabolism in addition to ageing, as the liver-specific deletion of SIRT6 in mice results in fatty liver formation.^8^ SIRT6 is a master regulator of glucose homeostasis and may have a large role in the treatments of metabolic diseases such as diabetes and obesity.^9^ Moreover, SIRT6 is a potent tumour suppressor and acts to suppress cancer metabolism.^10^

A previous study has demonstrated that SIRT6-deficient mice exhibits ~30% bone mineral density (BMD) loss and die at ~4 weeks of age. Rapid bone loss develops closely and synchronously with the accelerated ageing process, and such an osteoporotic phenotype is an excellent model for the study of ageing-associated osteoporosis.^3^ However, it remains unknown how SIRT6 regulates the progress of bone metabolism and how SIRT6 induces bone loss during the ageing progress. In this study, we used SIRTO-KO mice to investigate the bone pathological changes with ageing progression. Given the emerging roles of SIRT6 in anti-ageing, we demonstrated a smaller somatotype and decreased bone density in SIRT6-KO mice.

## Materials and methods

### Experimental animals and analysis of the skeletal phenotype

All mouse experiments were performed according to the approved protocols by the Institutional Animal Care and Use Committees at Sichuan University. SIRT6 global KO mice (129/SvJ) were purchased from Jackson Laboratory (Bar Harbor, ME, USA). Collagen type I α1-green fluorescent protein (ColIα1-GFP) transgenic mice were also purchased from Jackson Laboratory, and they expressed the topaz variant of green fluorescent protein (GFP) under the control of rat ColIα1 promoter/enhancer sequence. We crossed ColIα1-GFP mice with heterozygous SIRT6 mice several times until homozygosity for ColIα1-GFP was achieved, and GFP expression in the bone correlated with new bone mass. We used male mice (3 weeks old) in our studies. For skeleton staining, 3-week-old mice were eviscerated, and the skin was carefully removed. After 18 h of fixation in 95% ethanol, samples were stained in an Alcian blue solution (150 mg Alcian blue, 800 mL 95% ethanol, and 500 mL acetic acid) for 24 h. Next, the animals were transferred to 2% KOH for 48 h. The skeletons were then stained in Alizarin red solution (30 mg·L^−1^ Alizarin red in 2% KOH) and cleared in 1% KOH/20% glycerol.

### Histology and bone histomorphometry

Decalcified and non-decalcified sections of bone were prepared as previously described.^11, 12^ Briefly, mice (2 weeks old) were injected subcutaneously with calcein (8 mg·kg^−1^; Sigma-Aldrich Biotechnology, St Louis, MO, USA) 4 days after they had received a xylenol orange (XO; 15 mg·kg^−1^; Sigma-Aldrich Biotechnology, St Louis, MO, USA) injection. Samples were collected when the mice were 3 weeks old. Next, the samples were fixed with 4% paraformaldehyde overnight at 4 °C. Non-decalcified bones were embedded in optimal cutting temperature (OCT) compound, and 8-μm-thick frozen sections were prepared. Decalcified bones were embedded in paraffin, and 5- to 7-μm-thick sections were prepared. For micro-computed tomography (μCT) analysis, we performed high-resolution μCT scanning (μCT50; Scanco, Bassersdorf, Zurich, Switzerland) to measure the morphological indices of the metaphyseal regions of the femur or lumbar vertebrae (L5–6; 3 weeks old).^12, 13^ μCT imaging analysis of the metaphyseal regions was performed with 70 slices (10 μm per slice). The threshold is was 150 mg·cm^−1^. The expression of SIRT6 was determined using immunohistochemistry, according to the manufacturer's instructions (Vector Laboratories, Burlingame, CA, USA).

### Enzyme-linked immunosorbent assay

Test samples consisted of fresh mouse plasma. Enzyme-linked immunosorbent assay kits were purchased for TRAP5b and osteocalcin (OCN) assays, and were performed according to the manufacturer's protocols (Catalogue No. SEA902Mu and SEA471Mu, Cloud Clone, Wuhan, China).

### Mouse bone marrow stromal cell culture

Primary bone marrow stromal cells (BMSCs) were cultured using bone marrow obtained from mouse femurs and tibia as previously described.^14^ Briefly, mouse bone marrow was flushed from the femora and tibia, and cultured in α-modified Eagle's minimal essential medium (α-MEM; Catalogue No. SH30265; Hyclone Laboratories, Logan, UT, USA), which was supplemented with 10% fetal bovine serum (Hyclone Laboratories, Logan, UT, USA) and 1% penicillin/streptomycin (Life Technologies, Gaithersburg, MD, USA). The cell density was ~20 × 10^6^ per mL. For osteogenic differentiation, cells were cultured in basal medium for 5 days and were then changed to “osteogenic differentiation medium” (α-MEM supplemented with 10^−8^ mol·L^−1^ dexamethasone, 8 mmol·L^−1^ β-glycerophosphate, and 50 μg·mL^−1^ ascorbic acid) for an additional 14 days.

For XO labelling, cells were cultured in medium with 40 mmol·L^−1^ XO overnight. Images of the cells were obtained using a fluorescence microscope (green for ColIα1-GFP, blue for 4′,6-diamidino-2-phenylindole (DAPI), and red for XO). For alkaline phosphatase (ALP) staining, the medium was discarded, and the cells were fixed and washed twice with phosphate-buffered saline. Next, the cells were stained with ALP staining solution, which consisted of naphthol AS-MX phosphate and fast red violet LB salt (Sigma-Aldrich Biotechnology, St Louis, MO, USA). Finally, the stained wells were washed twice with tap water and imaged. For Von Kossa and Van Gieson staining, cells were stained using Von Kossa staining solution and Van Gieson staining solution, independently. Finally, the stained wells were washed twice with tap water and imaged.

The osteoclast cultures were generated as previously described.^15^ Briefly, bone marrow haematopoietic cells were cultured in the presence of macrophage colony-stimulating factor (M-CSF; 10 μg·mL^−1^ for 2 days), and incubated with both M-CSF and receptor activator of nuclear factor-κB ligand (RANKL; 10 μg·mL^−1^) for 5 days. Next, the cell medium was discarded, and the cells were fixed for 5 min and then stained for tartrate-resistant acid phosphatase (TRAP). TRAP-positive cells with more than two nuclei were quantified as osteoclasts.

### Statistical analysis

The positively stained area was calculated using the Image-Pro Plus software (IPP version 6.0; Media Cybernetics, Carlsbad, CA, USA). All experimental results were obtained from at least four separate experiments. Student's *t*-test analysis of variance was performed on the data using SPSS version 16.0 (SPSS, Chicago, IL, USA). Values were considered significantly different if *P*<0.05.

## Results

### Endogenous expression of SIRT6 in bone tissue

**Figure 1:**
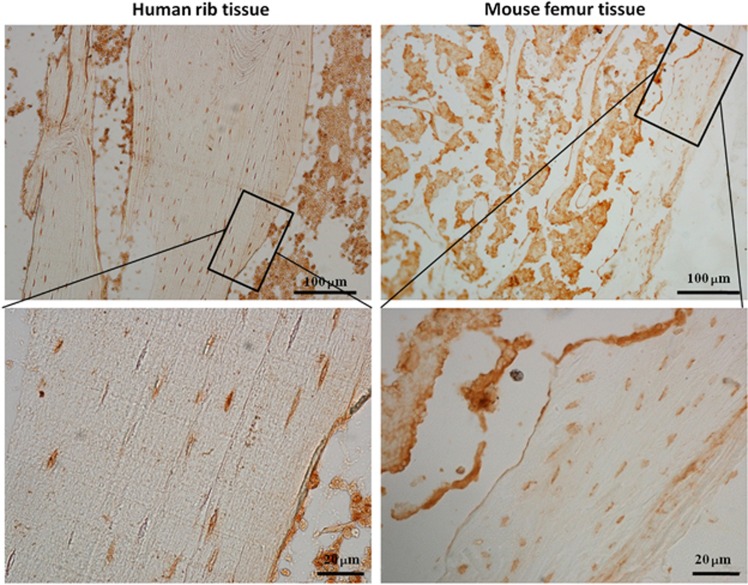
**Expression of SIRT6 in bone tissue of both mouse and human using immunohistochemistry staining.** The images are obtained from rib tissue of human and femur of wild-type mouse.

To investigate the basal expression of SIRT6 in bone tissue, we collected human rib bones and mouse femora, and examined these samples using paraffin section immunohistochemistry. SIRT6 was expressed in osteocytes, osteoblasts (along the surface of the mineralized tissue), and many types of marrow stroma cells of human rib bones, as well as the mouse femora (Figure 1). These results indicated that the positive expression of SIRT6 might participate in bone metabolism.

### Skeleton development analysis on SIRT6-KO mice

**Figure 2:**
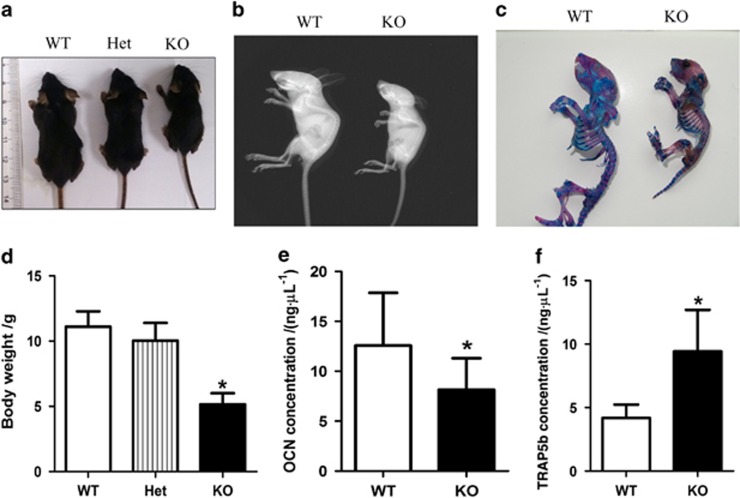
**SIRT6 knock out mice.** (**a**, **d**) Body weight was determined in ~9–11 mice for each group. (**b**) X-ray image. (**c**) Alizarin red S- and Alcian blue staining for skeletons. (**e**, **f**) Osteocalcin and TRAP5b concentration in blood was analyzed using enzyme-linked immunosorbent assay (ELISA) with ~11–12 mice in each group. *P<0.05. Het, heterozygote; KO, knock out; OCN, osteocalcin; WT, wild type.

We successfully bred and divided male littermate mice at the age of 3 weeks into groups consisting of wild type (WT), SIRT6 heterozygote and SIRT6-KO mice according to genotype identification analysis. On the basis of previous research on ageing SIRT6-KO mice,^3^ the entire body skeleton was investigated in this study. Overall, SIRT6-KO mice exhibited remarkable dwarfism and weight loss compared with their WT littermates, but no significant difference was observed between WT and SIRT6 heterozygote mice (Figure 2a–2d). Furthermore, Alizarin red S- and Alcian blue staining was performed to observe alterations in the entire body skeleton structure (Figure 2c). Compared with the WT littermates, cartilage bone shown as blue staining in SIRT6-KO mice was significantly decreased. In addition, lower calcification of the long bone was also observed when using red staining in SIRT6-KO mice. Moreover, X-ray analysis for the skeleton indicated that the whole-body BMD of SIRT6-KO mice was decreased, which was consistent with the findings obtained in previous work.^3^ Furthermore, to investigate the condition of whole-body bone (Figure 2b) metabolism, we collected serum from WT and SIRT6-KO mice to examine the expression of some markers of bone remodelling. The OCN (a marker of bone mass) concentration in serum was significantly decreased, whereas the TRAP5b concentration was significantly increased (Figure 2e and 2f), which indicated bone mass loss and bone resorption increase. Overall, SIRT6 deficiency in mice resulted in skeletal dysplasia, BMD decrease, and bone resorption increase of whole body bone tissue.

**Figure 3:**
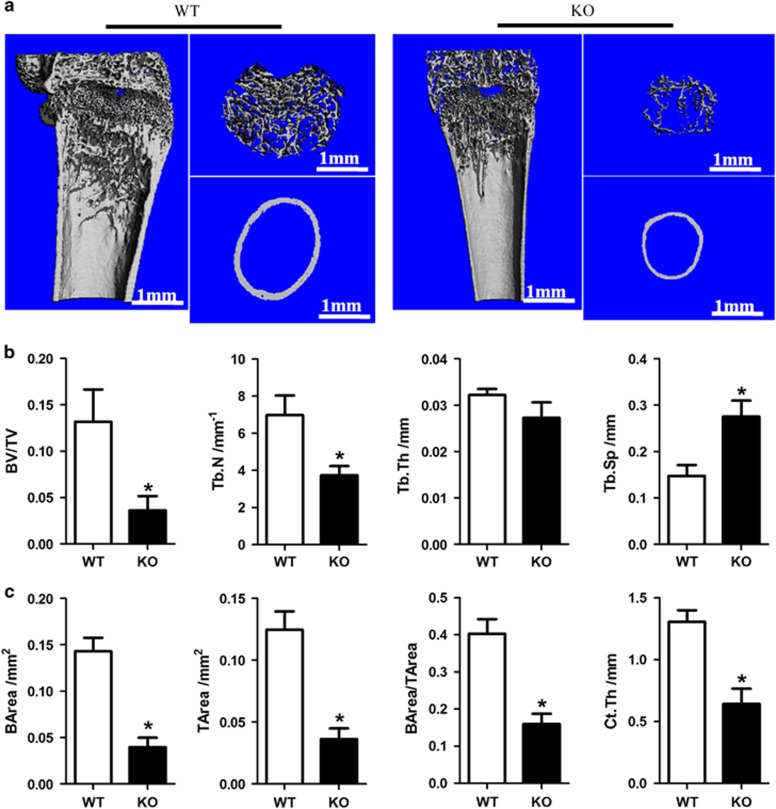
**Micro-CT analysis of trabecular and cortical bone of the femur.** (**a**) Femur tissue. Trabecular bone is from the part nearby the metaphysical area. Cortical bone is from mid-shaft. (**b**, **c**) Representative parameters of trabecular and cortical bone, respectively. *P<0.05. There were seven mice in each group used for the statistical analyses. BArea, bone area; BV/TV, bone volume/the total volume; CT, computed tomography; Ct.Th, cortical bone thickness; KO, knock out; TArea, the total area; Tb.N, trabecular number; Tb.Sp, trabecular separation; Tb.Th, trabecular thickness; WT, wild type.

**Figure 4:**
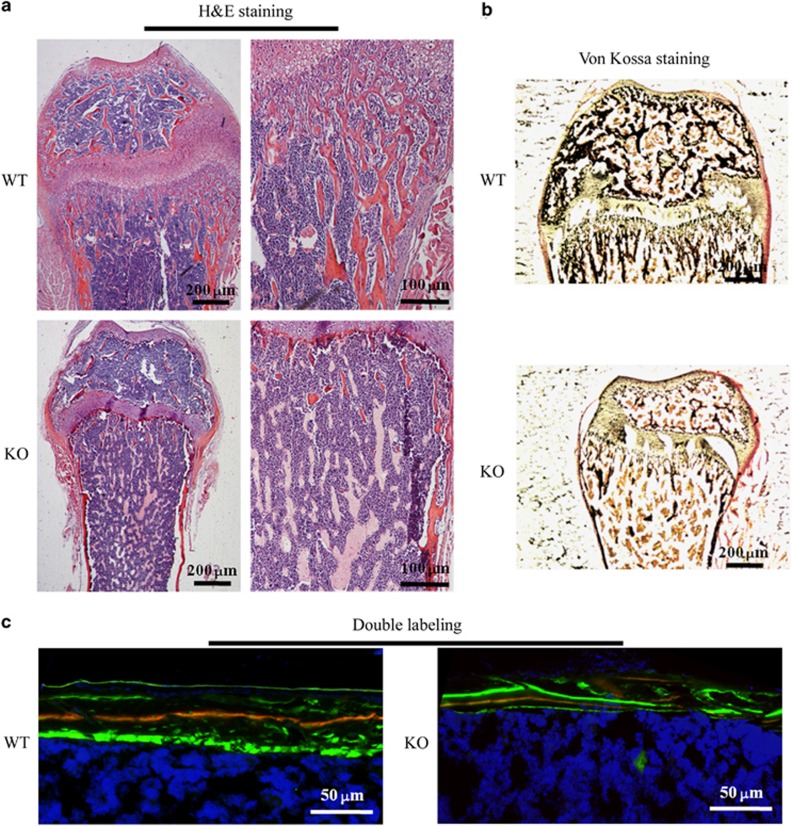
**Histological examination.** (**a**) Haematoxylin and eosin staining of the femur nearby the metaphysic area. (**b**) Van Gieson (red) and Von Kossa (black) staining for the mineralization of bone tissue. (**c**) Double-labelling analysis for bone formation at the femoral mid shaft. The red line is marked using xylenol orange, while the green line is marked by calcein. H&E, haematoxylin and eosin; KO, knock out; WT, wild type.

Bone histomorphometry was performed using μCT analysis, which further demonstrated bone growth retardation and severe osteopenia in SIRT6-KO mice. Trabecular bone mass formation was significantly impaired, with a reduced trabecular bone volume per tissue volume and a reduced trabecular number. The trabecular space was concomitantly increased (Figure 3a and 3b). Moreover, the cortical bone thickness of SIRT6-KO mice was decreased, as observed in the longitudinal and transverse mid-diaphysis sections. The parameters of cortical bone, cortical bone area, total cross-sectional area, cortical area fraction (BArea/TArea), and average cortical thickness were all decreased (Figure 3a–3c). However, histological examination showed a size difference in the femora of SIRT6-KO mice. A significantly reduced trabecular bone mass near the femur growth plate was demonstrated in haematoxylin and eosin (H&E) staining sections. Furthermore, the diaphyseal cortical bone was thinner than that of WT mice (Figure 4a). Von Kossa staining presented less calcium deposition with less black mineralized bone tissue in the SIRT6-KO mouse femora (Figure 4b). Double fluorochrome labelling revealed a reduction in the distance between green (stained by calcein) and red (stained by XO) in SIRT6-KO mice, which indicated a lower bone formation rate and was suggestive of osteogenic activity (Figure 4c). These results presented severely impaired bone metabolism due to SIRT6 KO, which is a clear symptom of osteoporosis.

### Osteogenesis in SIRT6-KO mice

**Figure 5:**
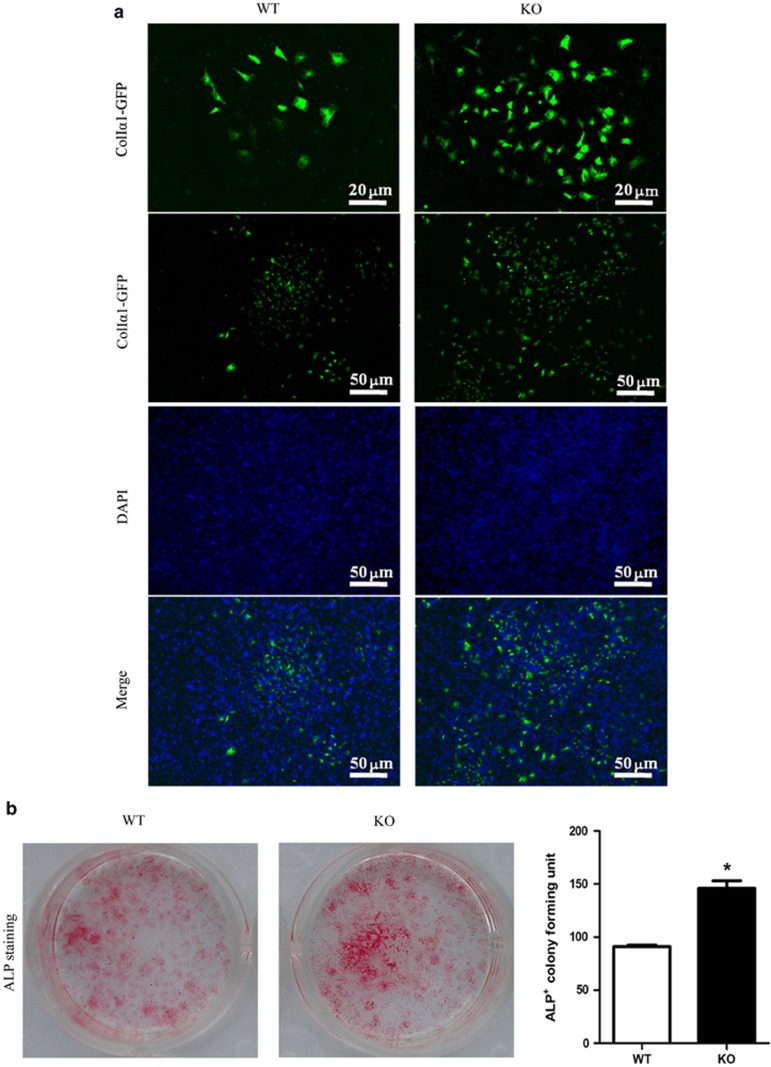
**Day 7 of cell culture for osteoblastogenesis from bone marrow stromal cells.** (**a**) ColIα1-GFP expression. (**b**) ALP for the cell colony forming units. The data are representative of six independent experiments. *P<0.05. ALP, alkaline phosphatase staining; DAPI, 4′,6-diamidino-2-phenylindole; GFP, green fluorescent protein; KO, knock out; WT, wild type.

**Figure 6:**
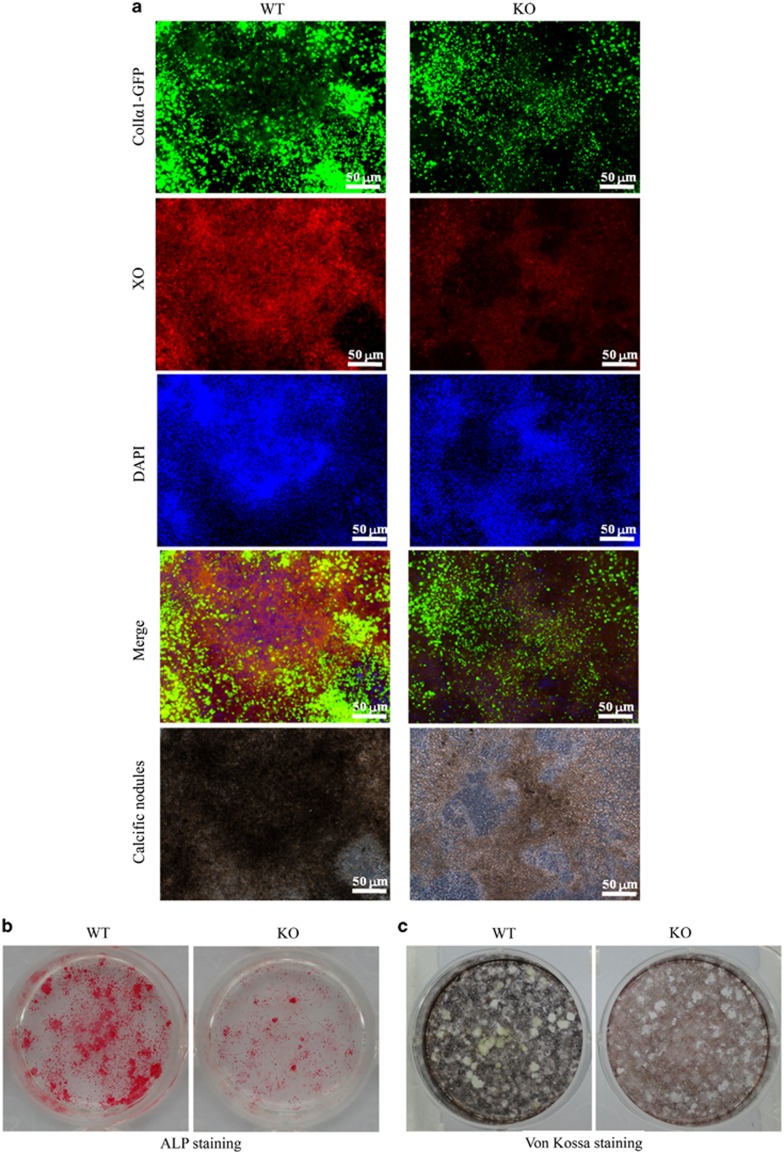
**Day 21 of cell culture for osteoblastogenesis.** (**a**) Expression of ColIα1-GFP, xylenol orange fluorescence for calcification and calcific nodules condition. (**b**, **c**) Alkaline phosphatase staining and Von Kossa staining. ALP, alkaline phosphatase; DAPI, 4′,6-diamidino-2-phenylindole; GFP, green fluorescent protein; KO, knock out; WT, wild type; XO, xylenol orange.

To further investigate whether SIRT6 directly regulates bone formation, we cultured primary BMSCs for osteogenesis *in vitro* (Figures 5 and 6). Interestingly, ALP production, which is an early marker of osteogenesis, was slightly increased at an early stage of osteogenesis (Figure 5). However, it was markedly decreased during the late stage of osteogenesis at day 21 (Figure 6). Similarly, ColIα1-GFP was observed with increased expression at day 7, but markedly decreased expression was observed at day 21. Fewer calcified nodules were observed using phase contrast microscopy in SIRT6-KO BMSCs. This condition of mineralization was also detected using XO and Von Kossa staining simultaneously, which further demonstrated significantly decreased osteogenesis. These results, which were consistent with our observations *in vivo,* revealed that SIRT6 could directly regulate bone formation.

### Osteoclastogenesis in SIRT6-KO mice

**Figure 7:**
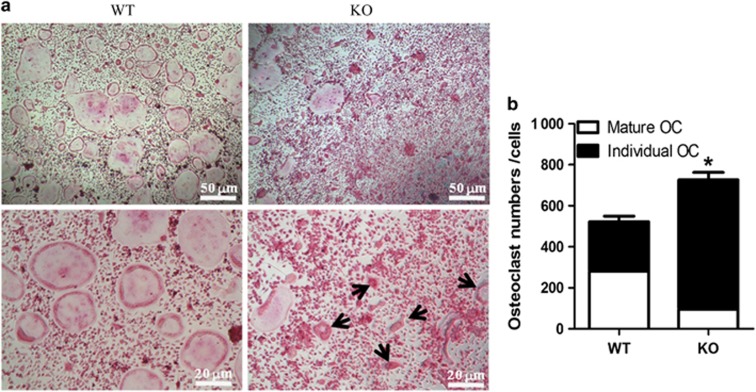
**Day 7 of cell culture for osteoclastogenesis.** (**a**) Trap staining for osteoclast. (**b**) The cell number of osteoclast per well. The 48-well plates were used for cell culture. Large cells with multi-nuclei and foamy cytoplasm were quantified as mature osteoclasts. Other Trap+ but smaller cells with more than two nuclei were marked as individual osteoclasts, such as the cells indicated by black arrows in figure. The data are representative of six independent experiments. *P<0.05. KO, knock out; OC, osteoclast; Trap, tartrate-resistant acid phosphatase; WT, wild type.

Osteoclastogenesis is also an important aspect of bone metabolism.^6, 16^ Thus, we wondered whether SIRT6 directly regulated osteoclastogenesis. We cultured primary BMSCs from WT and SIRT6-KO mice that were stimulated with M-CSF and RANKL for osteoclast induction. On day 7, TRAP staining revealed that there were fewer calcified mature giant osteoclasts with multi-nuclei and foamy cytoplasm in the SIRT6-KO group. However, a significant increase in TRAP+ individual osteoclasts with more than two nuclei was observed in SIRT6-KO osteoclasts (Figure 7). We proposed that cell fusion at the late stage of osteoclast formation might have been disrupted by SIRT6 deficiency, thereby inhibiting the maturation process. Taken together, these results indicated that SIRT6 has a role in directly controlling the formation, morphology, and number of mature osteoclasts.

## Discussion

In this study, SIRT6-KO mice were proven to be a good model for the study of SOP disease. It has been reported that SIRT6-KO mouse is an ageing model.^3, 17^ On the basis of this research, we aimed to fully investigate the phenotype of bone metabolism in detail and demonstrate its involvement in severe osteoporosis, which is ageing-related.

Generally, bone formation begins in mesenchymal cells from the bone marrow, which differentiate into osteoblasts that are attached to the bone surface and, consequently, differentiate into mature osteocytes, which are buried inside the calcified tissue.^6, 18^ Basal expression of SIRT6 was found in bone marrow and mature osteocytes in both mouse and human models (Figure 1), thereby suggesting that SIRT6 is likely to participate in the entire process of bone formation. The deterioration in both body length and weight and the reduction of cartilage and mineralized bone (Figure 2) were similar to the manifestation of osteopenia in aged people. Trabecular bone in the metaphyseal area and cortical bone in the diaphysis was reduced, as shown in μCT analyses, and converted into a status that appears weaker than the normal (Figure 3). Scattered trabecular bone detected using H&E and Von Kossa analysis revealed that bone metabolism of SIRT6-KO mice was disrupted, which was also shown by a lower bone formation rate (Figure 4) and by markers that were altered in blood, indicating whole-body bone metabolism (Figure 2). To a greater degree, most of the detailed phenotypes of osteopenia caused by SIRT6 KO are presented in our work, which describes the symptom of SOP disease.

We further investigated SOP at the cellular level *in vitro* to identify SOP results from weakened osteogenesis and enhanced bone resorption caused by SIRT6 KO directly. Bone-related features are an integrative effect that needs to be differentiated into osteogenesis and bone resorption.^1, 19^ ELISA of the blood of SIRT6-KO mice revealed decreased OCN and increased TRAP5b (Figure 2), which demonstrated that bone activity featuring SIRT6 deficiency was affected by both bone formation and bone resorption. Thus, bone loss in SIRT6-KO mice resulted from an imbalance of osteogenesis and bone resorption. Double labelling of bone revealed a conspicuous performance of sluggish new bone growth progression (Figure 4). Isolated and differentiated osteoblasts were weak in collagen formation and calcification, as shown in Figure 5, whereas isolated and differentiated osteoclasts were strong in bone resorption, as shown in bone slices (Figure 7). With less new bone formed and more bone resorbed in SIRT6-KO mice, the balance of the bone condition tilts naturally to bone loss.

Interestingly, osteogenic induction experiments showed a slight increase in ALP and ColIα1-GFP in BMSCs at the early stage of 7 days, whereas both decreased and the staining was reduced at the late stage (Figure 6). Sugatani *et al.*^20, 21, 22^ obtained similar results with SIRT6-KO BMSCs, which may be explained by the increased expression of Runx2 in osteoblasts.^20, 21, 22^ In all, SIRT6 defects further support the idea that SIRT6 leads to low calcification and exacerbates senile osteoporosis to some degree. Furthermore, we are interested in continuing our research to obtain more detailed evidence on the mechanism of SOP disease with SIRT6 deficiency.
